# Multimorbidity and long-term care dependency—a five-year follow-up

**DOI:** 10.1186/1471-2318-14-70

**Published:** 2014-05-28

**Authors:** Daniela Koller, Gerhard Schön, Ingmar Schäfer, Gerd Glaeske, Hendrik van den Bussche, Heike Hansen

**Affiliations:** 1 Centre for Social Policy Research, University of Bremen, Mary-Somerville-Straße 5, 28359 Bremen, Germany; 2 Department of Medical Biometry and Epidemiology, University Medical Center Hamburg-Eppendorf, Martinistr 52, 20246 Hamburg, Germany; 3 Department of Primary Medical Care, University Medical Center Hamburg-Eppendorf, Martinistr 52, 20246 Hamburg, Germany

**Keywords:** Long-term care, Multimorbidity, Claims data, Cox regression

## Abstract

**Background:**

Not only single, but also multiple, chronic conditions are becoming the normal situation rather than the exception in the older generation. While many studies show a correlation between multimorbidity and various health outcomes, the long-term effect on care dependency remains unclear. The objective of this study is to follow up a cohort of older adults for 5 years to estimate the impact of multimorbidity on long-term care dependency.

**Methods:**

This study is based on claims data from a German health insurance company. We included 115,203 people (mean age: 71.5 years, 41.4% females). To identify chronic diseases and multimorbidity, we used a defined list of 46 chronic conditions based on ICD-10 codes. Multimorbidity was defined as three or more chronic conditions from this list. The main outcome was “time until long-term care dependency”. The follow-up started on January 1^st^, 2005 and lasted for 5 years until December 31^st^, 2009. To evaluate differences between those with multimorbidity and those without, we calculated Kaplan–Meier curves and then modeled four distinct Cox proportional hazard regressions including multimorbidity, age and sex, the single chronic conditions, and disease clusters.

**Results:**

Mean follow-up was 4.5 years. People with multimorbidity had a higher risk of becoming care dependent (HR: 1.85, CI 1.78–1.92). The conditions with the highest risks for long-term care dependency are Parkinson’s disease (HR: 6.40 vs. 2.68) and dementia (HR: 5.70 vs. 2.27). Patients with the multimorbidity pattern “Neuropsychiatric disorders” have a 79% higher risk of care dependency.

**Conclusions:**

The results should form the basis for future health policy decisions on the treatment of patients with multiple chronic diseases and also show the need to introduce new ways of providing long-term care to this population. A health policy focus on chronic care management as well as the development of guidelines for multimorbidity is crucial to secure health services delivery for the older population.

## Background

Increased life expectancy and the aging of the baby-boom generation will lead to a higher number of older adults [1], and this comes with the need to manage chronic conditions that are more common in older age. One of the main challenges is and will be the management of multiple chronic conditions [2-4]. Multimorbidity is becoming the normal situation rather than the exception in the older generation [2,5-7]. But while the number of scientific papers focusing on multiple chronic conditions has increased significantly during recent decades [5,8], treatment guidelines still focus on single diseases and do not capture the complexity of multiple chronic diseases or consider possible prioritization of treatment options [9-14].

Compared with single diseases, multimorbidity has been shown to have a negative impact on a person’s health [15] and on the continuity of primary care [16]. People with multimorbidity die earlier [17-19], even if this correlation is not always clear [5,20,21]. (Multiple) chronic diseases are also known to be prevalent, especially in nursing homes [22,23]; however, the impact of these conditions on long-term care dependency remains unclear. Previous studies have shown a correlation between multimorbidity and functional impairment [9,21,24,25], but the definition of functional impairment and of long-term care dependency differs between studies [6]. Also, the definition of multimorbidity varies greatly between studies. In this study, multimorbidity is the co-existence of at least three chronic conditions over a time period of at least 1 year (see Methods and Discussion).

In the German statutory insurance system, long-term care insurance exists parallel to statutory health insurance, covering both institutionalized and ambulatory long-term care services. Health insurance is mandatory in Germany. In 2013, the majority of the population is insured in one of the 134 statutory health insurance schemes; 11% are covered by private health insurance [26]. A person can apply for long-term care coverage and will be evaluated based on their performance in activities of daily living (ADL). If care dependency is shown, the amount paid by the insurance depends on the severity of dependency (rated on three levels). The level of care is determined by an expert rating at the home of the applicant and can be re-evaluated at a later point in time. Until a person is assigned to a care dependency level, he or she cannot make claims for long-term care insurance.

The objective of this study is to follow up a cohort of older adults over 5 years with the end-point “long-term care dependency”. In this way, we are looking to answer the following questions:

● Over a time period of 5 years, what impact does multimorbidity have on long-term care dependency?

● What specific chronic conditions and disease clusters are related to long-term care dependency and do they differ for different age groups and between men and women?

## Methods

### Design and study population

This study was based on claims data from a German statutory health insurance company. Our cohort is based on data from the Gmünder ErsatzKasse (GEK, now called BARMER GEK after a fusion of two sickness funds in 2010), which insured 1.7 million people located in all regions of Germany (about 2% of the German population). We selected all those who were insured throughout the year 2004 and were at least 65 years old at that time (N = 123,224). Pseudonymized data were available, so a personal level analysis was possible. The data included beneficiary information on insurance times, age and sex, as well as diagnoses made in the ambulatory sector and claims made to long-term care insurance.

To identify chronic conditions and multimorbidity, we defined a list of 46 chronic conditions based on ICD-10 codes (see Table 1).

**Table 1:** List of 46 chronic conditions based on ICD-10 codes

**No.**	**Chronic condition**	**ICD-10 codes**
1	Hypertension	I10–I15
2	Lipid metabolism disorders	E78
3	Chronic low back pain	M40–M45, M47, M48.0–M48.2, M48.5–M48.9 M50–M54
4	Severe vision reduction	H17–H18, H25–H28, H31, H33, H34.1–H34.2, H34.8–H34.9, H35–H36, H40, H43, H47, H54
5	Joint arthrosis	M15–M19
6	Diabetes mellitus	E10–E14
7	Chronic ischemic heart disease	I20, I25, I21
8	Thyroid diseases	E01–E05, E06.1–E06.3, E06.5, E06.9, E07
9	Cardiac arrhythmias	I44–I45, I46.0, I46.9, I47–I48, I49.1–I49.9
10	Obesity	E66
11	Hyperuricemia/gout	E79, M10
12	Prostatic hyperplasia	N40
13	Lower limb varicosis	I83, I87.2
14	Liver disease	K70, K71.3–K71.5, K71.7, K72.1, K72.7, K72.9, K73–K74, K76
15	Depression	F32–F33
16	Asthma/COPD	J40–J45, J47
17	Gynecological problems	N81, N84–N90, N93, N95
18	Atherosclerosis/PAOD	I65–I66, I67.2, I70, I73.9
19	Osteoporosis	M80–M82
20	Renal insufficiency	N18–N19
21	Cerebral ischemia/chronic stroke	I60–I64, I69, G45
22	Cardiac insufficiency	I50
23	Severe hearing loss	H90, H91.0, H91.1, H91.3, H91.8, H91.9
24	Chronic cholecystitis/gallstones	K80, K81.1
25	Somatoform disorders	F45
26	Hemorrhoids	I84
27	Intestinal diverticulosis	K57
28	Rheumatoid arthritis/chronic polyarthritis	M05–M06, M79.0
29	Cardiac valve disorders	I34–I37
30	Neuropathies	G50–G64
31	Dizziness	H81–H82, R42
32	Dementia	F00–F03, F05.1, G30, G31, R54
33	Urinary incontinence	N39.3–N39.4, R32
34	Urinary tract calculi	N20
35	Anemia	D50–D53, D55–D58, D59.0–D59.2, D59.4–D59.9, D60.0, D60.8, D60.9, D61, D63–D64
36	Anxiety	F40–F41
37	Psoriasis	L40
38	Migraine/chronic headache	G43, G44
39	Parkinson’s disease	G20–G22
40	Cancer	C00–C14, C15–C26, C30–C39, C40–C41, C43–C44, C45–C49, C50, C51–C58, C60–C63, C64–C68, C69–C72, C73–C75, C81–C96, C76–C80, C97, D00–D09, D37–D48
41	Allergy	H01.1, J30, L23, L27.2, L56.4, K52.2, K90.0, T78.1, T78.4, T88.7
42	Chronic gastritis/GERD	K21, K25.4–K25.9, K26.4–K26.9, K27.4–K27.9, K28.4–K28.9, K29.2–K29.9
43	Sexual dysfunction	F52, N48.4
44	Insomnia	G47, F51
45	Tobacco abuse	F17
46	Hypotension	I95

COPD, chronic obstructive pulmonary disease; PAOD, peripheral arterial occlusive disease; GERD, gastroesophageal reflux disease.

These chronic conditions were chosen on the basis of the ADT panel (where frequent chronic medical conditions in GP surgeries are described by the Central Research Institute of Statutory Ambulatory Health Care in Germany) and based on the prevalence in the GEK (for further details on cohort selection, see [27]).

We included all diagnoses made for each person for each quarter of the year 2004. A person was considered as chronically ill if he/she had a diagnosis from the chronic conditions list in three out of the four quarters. This rather strict inclusion criterion was chosen to avoid accidental diagnoses or acute diseases. If a person had at least three chronic conditions, he/she was considered as part of the multimorbidity sub-cohort.

### Statistical analyses

The main outcome was the time until a person made a claim for long-term care insurance. For this specific research question, all those who already had a care level in 2004 were excluded from the analysis. The follow-up started after the 1 year of cohort definition on January 1^st^, 2005 and lasted for 5 years until December 31^st^, 2009. People who died during this time or left the insurance scheme for other reasons were included in the analysis as censored cases. People were analyzed in the group they were defined in at the start of the observation period throughout the whole follow-up period.

To evaluate differences between those with multimorbidity and those without with respect to long-term care dependency, we first calculated Kaplan–Meier curves stratified for multimorbidity (yes/no). Furthermore, Cox proportional hazard regressions were performed to estimate hazard ratios (HR) and 95% confidence intervals (95% CI). The HR shows the rate at which the population is likely to get to an event compared with the control population. In our case, if multimorbidity had a HR of 2, the “chance” or “hazard” of becoming long-term care dependent was twice as high as for those without multimorbidity. We calculated four models to adjust stepwise for possible influencing variables. In the first model, we determined the HR for care dependency for multimorbidity and entered sex (two categories) and age (as a continuous variable) as control variables. As multimorbidity has a different effect on men and women in different age groups, we also included an interaction term in the analysis. In the second model, we added all chronic conditions to identify those conditions with a major impact on long-term care dependency.

To address the complexity of multimorbidity and its influence on long-term care dependency, we calculated two additional models: Model 3 includes three disease clusters that can be assigned to 50% of the patients and capture 75% of the variance in chronic diseases in this cohort. These patterns are: “Neuropsychiatric disorders” (NPS), “Cardiovascular and metabolic disorders” (CMD), and “Anxiety, depression, somatoform disorders and pain” (ADS/P). Patients are assigned to a pattern if they have at least three diseases from the pattern-specific morbidity spectrum. The patterns were identified by factor analysis in another study [28]. Model 4 includes the variable for multimorbidity and the top five single diseases with the highest risk for care dependency seen in Model 1. This model was included to determine whether multimorbidity has an independent effect on care dependency, irrespective of the single diseases.

The study was approved by the ethics committee of the Medical Association of Hamburg (PV3057). All analyses were performed using the Statistical Analysis Systems (SAS Release 9.3, SAS Institute Inc. Cary, NC, USA).

## Results

The main outcome was the time until a first claim was made for long-term care insurance. For this research question, all those who already had a care level in 2004 were excluded from the analysis. We also excluded people with inconsistencies in the continuity of their membership (i.e., if a person left the health insurance company for longer than 1 month). Finally, 115,203 people were included in the analysis. Basic information on the cohort is found in Table 2.

**Table 2:** Cohort information

	**Multimorbid**	**Not multimorbid**	**ALL**
N	66,384	48,819	115,203
(%)	57.62	42.38	
Men	56.49	61.51	58.62
Women	43.51	38.49	41.38
Age (mean) years	72.26	70.38	71.46
Age (mean) men	71.93	70.07	71.11
Age (mean) women	72.68	70.88	71.97
Diseased during follow-up	15.30	9.80	13.00
Mean follow-up	4.41	4.63	4.50

**Figure 1:**
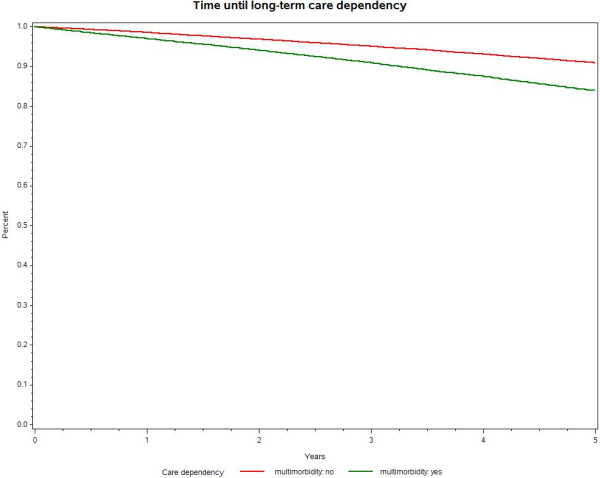
Kaplan–Meier curves for long-term care dependency by morbidity.

**Figure 2:**
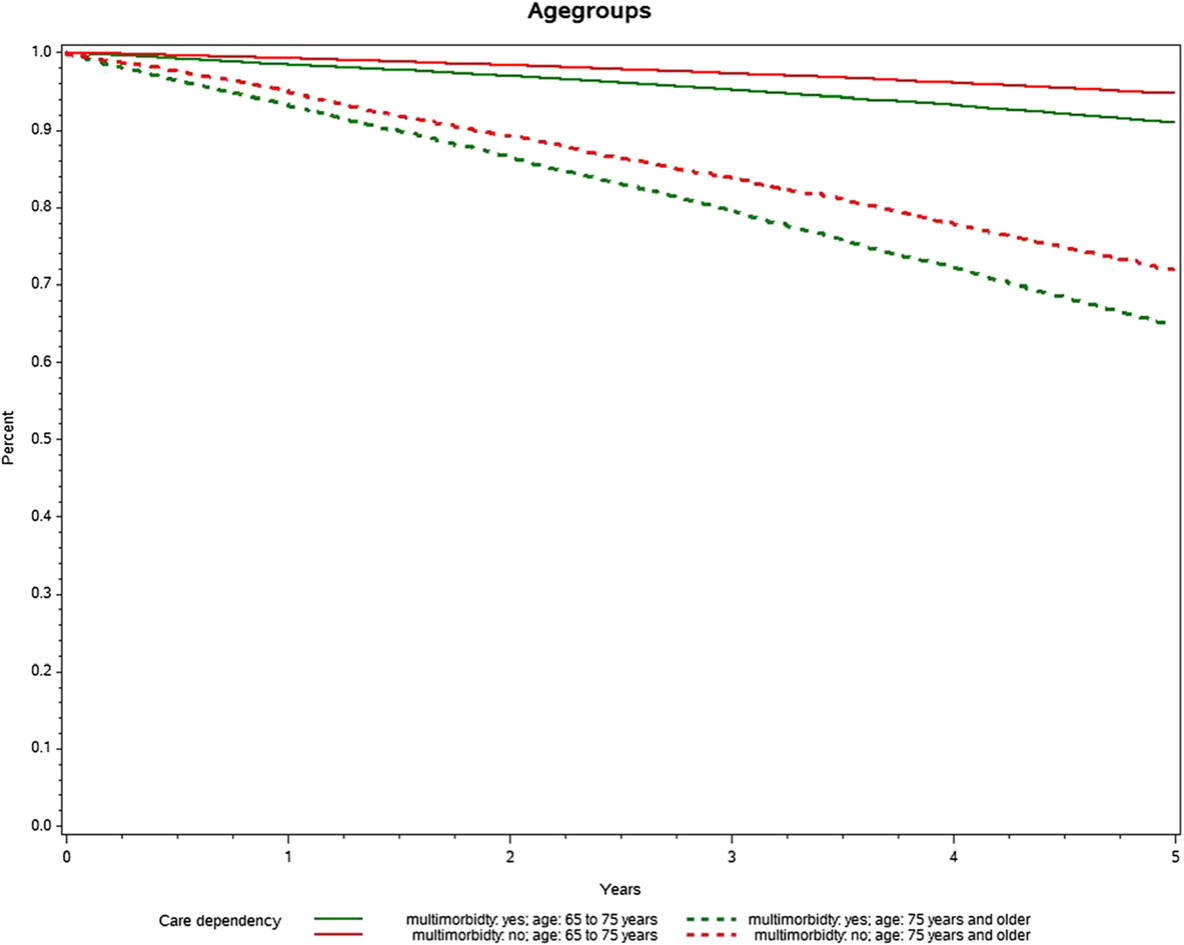
Kaplan–Meier curves for long-term care dependency by morbidity and age.

Mean follow-up was 4.5 years and was slightly shorter for insured people with at least three chronic conditions. They were also older by 2.1 years than the non-multimorbid sub-cohort. Although there were more men in both groups, the percentage of women was higher in the multimorbid sub-cohort; 13% of the cohort died during the follow-up period.Figure 1 shows the Kaplan–Meier curves for time until long-term care dependency for multimorbid vs. not multimorbid people. The graph clearly shows that those with at least three chronic conditions had a higher risk of becoming care dependent than non-multimorbid people during the follow-up period. A clear difference was already visible after the first year. After 5 years, 15.3% of those suffering from multimorbidity had become long-term care dependent compared with 8.7% of those without multimorbidity (p for log rank <0.0001).As long-term care dependency is likely to be correlated with age, we adjusted for a higher care dependency with higher age. Kaplan–Meier curves were calculated for multimorbid and non-multimorbid individuals stratified for age, grouping the cohorts in age groups 65–74 years and 75 years and older (see Figure 2).

**Figure 3:**
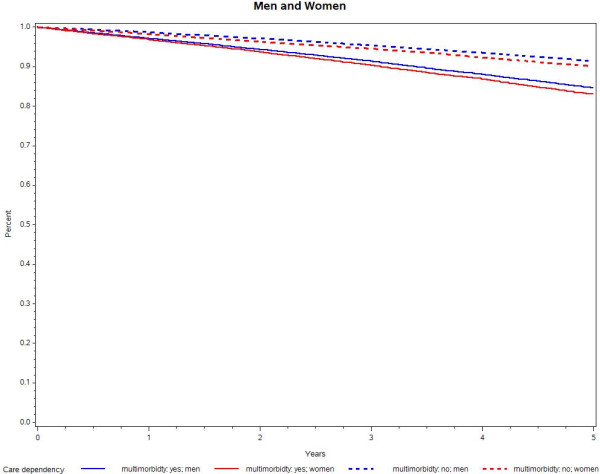
Kaplan–Meier curves for long-term care dependency by morbidity and sex.

Those with multimorbidity became care dependent more often than those with less than three chronic conditions, as seen before. However, older adults aged 75 years and older had a much higher risk of care dependency; after 5 years, 26.3% of the non-multimorbid people were care dependent compared with 32.5% of those with multimorbidity in the older age group.This analysis was also stratified for gender; the data show that women were more likely to become long-term care dependent over the 5-year observation period than men. However, the difference between women with fewer than three conditions and those with three or more was comparable to the difference between men with multimorbidity and those without (see Figure 3).

To show different influencing factors on long-term care dependency, we calculated four Cox proportional hazard models. The results are shown in Table 3. In the null model (including only multimorbidity), multimorbid patients had an 85% higher risk of becoming long-term care dependent than those with no multimorbidity (HR: 1.85, CI 1.78–1.92; data not shown). This influence remained significant when controlled for age, sex, and the interaction between both, but weakened noticeably to a HR of 1.41 in Model 1. This model was also run with a chronic disease count instead of the multimorbidity variable. With each additional chronic condition, the risk of becoming long-term care dependent increased by 6.4% (data not shown).

**Table 3:** Cox proportional hazard regression

	**Model 1**			**Model 2**			**Model 3**			**Model 4**		
	**HR**	**CI**		**HR**	**CI**		**HR**	**CI**		**HR**	**CI**	
Multimorbidity	1.41	1.36	1.47							1.16	1.12	1.21
Sex	0.37	0.25	0.54	0.51	0.35	0.75	0.43	0.3	0.62	0.47	0.32	0.68
Age	1.14	1.13	1.15	1.13	1.12	1.14	1.14	1.13	1.15	1.14	1.13	1.15
Interaction age/sex	1.01	1.01	1.02	1.01	1.00	1.01	1.01	1.01	1.02	1.01	1.01	1.02
Cluster: CMD*							1.11	1.07	1.15			
Cluster: ADSP*							1.25	1.21	1.30			
Cluster: NPS*							1.79	1.63	1.97			
Parkinson’s disease				2.86	2.59	3.15				3.04	2.76	3.35
Dementia				2.37	2.18	2.57				2.49	2.29	2.70
Tobacco abuse				1.69	1.46	1.96				1.86	1.60	2.15
Cerebral ischemia/chronic stroke				1.53	1.44	1.63				1.53	1.44	1.63
Diabetes mellitus				1.48	1.42	1.54				1.51	1.46	1.57
Urinary incontinence				1.39	1.27	1.52						
Cancer				1.36	1.30	1.42						
Cardiac insufficiency				1.32	1.26	1.40						
Asthma/COPD				1.32	1.26	1.39						
Anemia				1.30	1.19	1.43						
Depression				1.28	1.21	1.35						
Renal insufficiency				1.26	1.17	1.36						
Atherosclerosis/PAOD				1.22	1.15	1.28						
Rheumatoid arthritis/chronic polyarthritis				1.21	1.09	1.33						
Neuropathies				1.17	1.10	1.25						
Dizziness				1.15	1.06	1.24						
Liver disease				1.12	1.06	1.20						
Obesity				1.11	1.04	1.18						
Chronic ischemic heart disease				1.08	1.04	1.13						
Osteoporosis				1.08	1.02	1.15						
Joint arthrosis				1.08	1.03	1.13						
Insomnia				1.07	0.99	1.15						
Cardiac arrhythmias				1.06	1.01	1.12						
Cardiac valve disorders				1.06	0.97	1.15						
Hyperuricemia/gout				1.04	0.99	1.10						
Hypertension				1.02	0.98	1.05						
Hypotension				1.02	0.87	1.18						
Anxiety				1.01	0.88	1.16						
Chronic cholecystitis/gallstones				1.00	0.93	1.07						
Psoriasis				1.00	0.88	1.13						
Severe hearing loss				0.97	0.89	1.06						
Lower limb varicosis				0.96	0.91	1.01						
Chronic gastritis/GERD				0.96	0.90	1.01						
Allergy				0.93	0.85	1.02						
Severe vision reduction				0.92	0.88	0.96						
Chronic low back pain				0.90	0.87	0.94						
Migraine/chronic headache				0.90	0.78	1.03						
Intestinal diverticulosis				0.88	0.8	0.97						
Thyroid diseases				0.87	0.82	0.91						
Somatoform disorders				0.86	0.79	0.95						
Prostatic hyperplasia				0.84	0.79	0.89						
Urinary tract calculi				0.81	0.72	0.91						
Hemorrhoids				0.80	0.73	0.88						
Lipid metabolism disorders				0.79	0.76	0.82						
Sexual dysfunction				0.78	0.66	0.93						
Gynecological problems				0.75	0.68	0.83						

*Clusters: CMD: cardiovascular and metabolic disorders; ADS/P: anxiety, depression, somatoform disorders, and pain; NPS: neuropsychiatric disorders; see [28] for details.

In the second model, the results show those diseases with a high or low risk of leading to long-term care dependency. Those conditions with the highest risks for long-term care dependency were neurological disorders, mainly Parkinson’s disease and dementia. The risk of becoming long-term care dependent when having one of these two diseases was over twice as high as if the disease was not present. Stroke, diabetes, and oncologic diagnoses were also strongly associated with an increased risk of long-term care dependency. This effect could not be shown for conditions such as gynecological problems or lipid metabolism disorders; these diseases are very common and are not known to have a high impact on the activities of daily living (ADL).

In the third model, we included four disease clusters that are most prevalent in this cohort to address the complexity of multimorbidity in the sample. When controlled for age, sex, and the interaction term age*sex, all three clusters showed a significant correlation with long-term care dependency. Those in the CMD cluster had an 11% higher risk of becoming care dependent, ADS/P patients 25%, and patients in the NPS cluster had a 79% higher risk of care dependency within the follow-up time.

To determine whether multimorbidity itself has an effect on care dependency irrespective of single diseases, we included an additional model with multimorbidity as the independent variable, controlling for age, sex, the interaction term, and the top five conditions with the highest HR for care dependency found in Model 2 (Parkinson’s disease, dementia, tobacco abuse, cerebral ischemia/chronic stroke, diabetes mellitus). The effects of the single diseases are stronger than those seen in Model 2. Multimorbidity itself still has an effect; multimorbid patients still have a 16% higher risk of becoming care dependent even when controlled for the five diseases most associated with care dependency.

## Discussion

We found that those with multiple chronic conditions had a significantly higher risk of becoming long-term care dependent in a 5-year period. Specific diseases showed a strong impact on care dependency, namely dementia and Parkinson’s disease. This correlation between dementia and functional dependency/long-term care dependency is in line with previous results [24,29-31]. Stroke is also strongly correlated with care dependency; our results therefore point to a higher influence of neurological diseases on care dependency, compared with another study highlighting the influence of cerebrovascular disease, arthritis, and coronary artery disease [32]. Then again, neither dementia nor Parkinson’s disease was evaluated in that study.

This influence of neuropsychiatric diseases is also reflected in the high HR for patients in the NPS disease cluster, which includes dementias and Parkinson’s disease, but also other related diseases such as depression, stroke, and urinary incontinence [28]. We were able to show that, with every additional disease, the risk of becoming long-term care dependent increased by over 6% over the 5-year period. Marengoni and Angleman also showed a higher proportion of people with disability with more diseases, ranging from 4% with no condition to 28%of those with four or more [24], but higher numbers of chronic conditions were not distinguished. Our analysis also showed that all three of the identified clusters have a significant influence on care dependency.

As multimorbidity is associated with both age and sex [33], we included an interaction term in our analysis as a control variable. This was necessary to control for the fact that the pattern of multimorbidity is different for men and women at different ages. For instance, for both men and women in the age group 65–74 years, the HR for becoming long-term care dependent is 0.4, but for people aged 75 years and older, it is 3.3 for men and 3.9 for women (data not shown). This interaction term does have a significant effect on long-term care dependency, independent of the single diseases or the number of diseases. However, this effect is rather low when controlled for other variables.

Multimorbidty per se showed an association with care dependency even when controlled for the diseases most associated with care dependency. However, we could only adjust for the top five diseases to avoid multicollinearity in the regression model.

A major concern about comparing other studies with our results is the different definition of dependency and also the different inclusion of diseases and definition of co- or multimorbidity. A 2003 review identified 13 different ways of defining co- or multimorbidity [34]—a number that has increased over the last 10 years. A more recent review from the European General Practice Research Network identified 132 different definitions with a large number of sub-specifications [35]. Another study also addressed methodological differences, concluding the strong influence of definition on the prevalence of multimorbidity [6].

The diseases that were seen to have no higher risk for care dependency are also not clinically related to ADL, such as allergies or sexual dysfunction. As decline in ADL is the only factor evaluated for care dependency, this result is not surprising. We decided to keep those factors in the analysis to account for a broader spectrum of multimorbidity. Even if those conditions have no direct impact on long-term care dependency, they can influence the patient’s life and possibly their health care utilization habits.

### Strengths and limitations

Our analysis is based on health insurance claims data. The GEK insured about 2% of the German population during our follow-up time. The GEK primarily insured craftsmen before all sickness funds were opened to everyone in 1996 through a German health reform. However, the proportion of insured men still exceeds that of women today; we therefore carried out the separate gender analysis that did not result in different outcomes. A generalization of the results to the overall population should still be done cautiously. Also, the data are collected for financial purposes and therefore do not include any variables on the socio-economic background of the insured persons, or medical information such as test results or information on the severity of disease. We also have no information on the living situation, informal caregivers, or formal support paid out-of-pocket. All these factors might influence the need for long-term care and might therefore lead to an earlier or later application for long-term care insurance.

Some people who were classified as not multimorbid might have developed additional conditions during follow-up. We still analyzed them in their original group in order to compare those groups over the whole time period. Therefore, the effect of multimorbidity on long-term care dependency might be underestimated.

However, we were able include a large sample of over 123,000 people irrespective of their physical or mental condition, their living environment, or life circumstances. We were also able to follow up 115,203 people without care dependency over the 5 years, even if they moved, were severely impaired, or were in an institution. So we barely face any selection bias, which can be especially challenging for survey studies including cognitively impaired patients [36]. We focused on long-term care dependency and not on institutionalization. Some of those receiving a care level might move directly to a nursing home, but this is not a necessity. The insurance also covers ambulatory long-term care such as home aid services. As we were able to analyze all ambulatory care data, we also included a large selection of chronic conditions. However, to avoid accidental diagnoses, we included only those patients with diagnoses in three out of four consecutive quarters. We might therefore even have underestimated the prevalence of multimorbidity.

## Conclusion

We have shown for the first time in a large German dataset the influence of multimorbidity, disease clusters, and single diseases on care dependency in older people. Multimorbidity has a major impact on care dependency irrespective of age, gender, and single diseases. In particular, neurological conditions and disease clusters are highly correlated with care dependency. The results show the importance of multiple chronic conditions in health care delivery for older patients and highlight the need to introduce new ways of providing care to this population. Especially in diseases with the highest risk for long-term care dependency such as dementia or chronic stroke, there is much potential for prevention of the need for long-term care.

To focus on chronic care management for patients with multiple chronic disorders in an ambulatory setting is crucial for future research and health policy decisions. This approach needs to be implemented at all levels of care from primary care to specialists, rehabilitation, and long-term care, and should also include other health professionals as multimorbidity is becoming more the norm than the exception. Based on previous research and our results, the development of specific guidelines focusing on those with multimorbidity is long overdue to strengthen treatment options for patients and thus guarantee a longer independent life.

## Competing interests

GG received funding from statutory health insurance companies for scientific studies, among them from the GEK. The other authors declare no competing interests.

## Authors’ contributions

DK and HH drafted the study; DK performed the analysis and wrote the manuscript. GS advised on the statistical analyses; IS, HvdB, and GG took part in cohort design and study concept. All authors commented on the draft and approved the final version of the manuscript.

## Pre-publication history

The pre-publication history for this paper can be accessed here:

http://www.biomedcentral.com/1471-2318/14/70/prepub
